# Changes in Coronary Care for Acute Myocardial Infarction over the Past Two Decades (2000–2023) in Kaunas, Lithuania

**DOI:** 10.3390/jcm15103963

**Published:** 2026-05-21

**Authors:** Lolita Sileikiene, Abdonas Tamosiunas, Karolina Marcinkeviciene, Daina Kranciukaite-Butylkiniene, Sarunas Augustis, Dalia Lukšienė, Jolita Kirvaitiene, Gintare Sakalyte, Ricardas Radisauskas

**Affiliations:** 1 Institute of Cardiology, Medical Academy, Lithuanian University of Health Sciences, 50103 Kaunas, Lithuania; abdonas.tamosiunas@lsmu.lt (A.T.); karolina.marcinkeviciene@lsmu.lt (K.M.); daina.butylkiniene@lsmu.lt (D.K.-B.); sarunas.augustis@lsmu.lt (S.A.); dalia.luksiene@lsmu.lt (D.L.); gintare.sakalyte@lsmu.lt (G.S.); ricardas.radisauskas@lsmu.lt (R.R.); 2 Department of Preventive Medicine, Faculty of Public Health, Medical Academy, Lithuanian University of Health Sciences, 50103 Kaunas, Lithuania; 3 Department of Family Medicine, Medical Academy, Lithuanian University of Health Sciences, 50103 Kaunas, Lithuania; 4 Department of Internal Medicine, Medical Academy, Lithuanian University of Health Sciences, 50103 Kaunas, Lithuania; 5 Department of Environment and Occupational Medicine, Faculty of Public Health, Medical Academy, Lithuanian University of Health Sciences, 50103 Kaunas, Lithuania; jolita.kirvaitiene@lsmu.lt

**Keywords:** acute myocardial infarction, coronary care, time to hospitalization, treatment methods, sex, age, Lithuania

## Abstract

**Background/Objectives**: Epidemiological studies over the first decades of the 21st century have reported a decrease in cardiovascular disease (CVD) morbidity and mortality. Changes in coronary care for acute myocardial infarction (AMI) over these years, including the COVID-19 pandemic period, have been less studied in Eastern and Central Europe. The study aimed to assess changes in coronary care—the time of medical assistance and treatment—for AMI patients over 2000–2023 in urban Kaunas residents aged 25–64. **Methods**: The data source was study cases from the Kaunas Ischemic Heart Disease Registry (Registry)—Kaunas city residents aged 25–64 years included in the Registry according to MONICA project protocol evaluation methodologies. Data were analyzed by sex and age group (25–54 and 55–64 years). Descriptive statistics (chi-square and z-score values) were used to evaluate the data; the significance level was *p* < 0.05. A logistic regression analysis was performed to assess the odds ratios of death within 28 days across six time periods. **Results**: The proportion of AMI patients hospitalized up to 2 h from the onset of pain accounted for about one-fifth of all hospitalized patients in 2000–2016, while in 2017–2023, it significantly decreased. In 2017–2023, compared with 2000–2004 and 2009–2016, significantly fewer men who developed AMI were hospitalized within the first 2 h of emergency presentation (*p* < 0.05). Over the whole study period, fewer women with AMI were hospitalized within the first 2 h of pain as compared to men (*p* < 0.05). There were no significant differences in time from pain onset to hospitalization between the age groups. At the same time, from 2009 to 2012, more young AMI patients were hospitalized within the first 2 h (*p* < 0.05). Percutaneous coronary angioplasty (PTCA) with stenting (PCI) increased 30 times from 2000–2004 to 2020–2023. PCI has been the most available treatment for men with AMI since 2009 and stayed stable from 2013 (66.0%) until 2023 (72.1%). Women with AMI tended to get less PCI, PTCA, and coronary artery bypass grafting (CABG) than men. The pre-pandemic and COVID-19 periods did not differ in the proportions of reperfusion treatment methods used in both men and women. Thrombolysis was very rare, and since 2017, it has not been used in Kaunas because PCI has become more accessible. PCI (2000–2016) and CABG (2009–2016) were more prevalent among the 25–54-year-old AMI patients (*p* < 0.05). From 2017 to 2023, there were no differences between age groups in the reperfusion procedures used, nor were there differences in treatment between these groups during the pre-pandemic (2017–2019) and peri-COVID-19 pandemic (2020–2023) periods. **Conclusions**: In Kaunas, the treatment of patients with AMI has improved significantly over the past 20 years. The use of PCI has increased greatly, and the rate of CABG surgery stayed stable, while only every fifth patient has been admitted to the hospital in a timely manner. Men were more likely to receive PCI, and older patients were more likely to undergo CABG. Compared to the period of 2000–2004, the chance of dying within 28 days after AMI was significantly lower in 2017.

## 1. Introduction

Ischemic heart disease (IHD) remains the primary contributor to excessive global mortality from non-communicable diseases (NCDs), with a substantial increase in mortality observed since 2000 [1,2,3,4]. Epidemiological studies over the first decades of the 21st century have reported a decrease in cardiovascular disease (CVD) morbidity and mortality, though in Europe, CVDs continue to be the leading cause of death, accounting for approximately 4.1 million deaths annually and affecting over 60 million individuals, with notable geographic, socioeconomic, and sex-based disparities [5,6,7]. This burden is particularly pronounced in Eastern and Central Europe, where mortality rates remain considerably higher than in Western and Northern regions, largely reflecting disparities in risk factor prevalence and healthcare systems [7]. Some studies showed that case fatality declined in some European countries, while other investigators observed stable case fatality from acute myocardial infarction (AMI) [8]. These trends have been mainly attributed to the advancements introduced into daily clinical practice both in the prevention and in the treatment of AMI, such as timely reperfusion strategies, novel therapies, and intensified treatment for the management of modifiable cardiovascular risk factors [9].

Mortality from CVD in Lithuania is still one of the highest in Europe [7]. The leading cause of death in Lithuania over the past few decades has been CVD. In 2024, mortality from diseases of CVD was 658.7 per 100,000 population [10,11]. In 2024, deaths from CVD accounted for 56.8% of all deaths in women, and 44.6% in men, of which 60.4% died from IHD [11].

AMI refers to those manifestations that occur extremely suddenly and have a treatment window measured in minutes to hours, but which may be significantly mitigated by timely, short-term treatment [12]. In patients who have had AMI, reperfusion therapy should be applied as early as possible from the onset of symptoms; the timing of treatment reflects the efficiency and quality of the system that cares for patients suspected of having AMI [13].

Thus, provision of quality healthcare from the start of pain or recognition of the emergency until restoring blood flow in blocked coronary vessels is critical, and ongoing revision of processes and their outcomes while providing help to the AMI patients is required to develop adequate and supportive citizen and emergency assistance [7].

Changes in coronary care for AMI over the last decades, including the COVID-19 pandemic period, which impaired coronary care and accessibility to the healthcare system, have been less studied in Eastern and Central Europe.

The study aimed to assess changes in coronary care—the time of medical assistance and treatment—for AMI patients over the 2000–2023 period in urban Lithuanian citizens, based on data from the Kaunas Ischemic Heart Disease Registry.

## 2. Materials and Methods

### 2.1. Study Sample

This study analyzed the data collected by the Kaunas Ischemic Heart Disease Registry, describing the time parameters of medical assistance provided and the characteristics of treatment for individuals with AMI. According to the MONICA study protocol, the Kaunas ICH Registry is conducted using the “cold” method, i.e., the medical documentation of cases is reviewed after the individual has left the healthcare facility [14]. During the implementation of the Kaunas 25–64-year-old population IHD registry, cases were reviewed and selected from the statistical cards of persons discharged from inpatient care (Form No. 066/a-LK)—the disease codes from The International Classification of Diseases, Tenth Revision (ICD-10) I20.0, I21–I22, or deaths with ICD-10 codes I20-I25 have been included. According to the MONICA protocol, other possible ICD-10 codes, such as I10–I15, I26–I28, I30–I52, I70–I79, E10–E14, E66, E78, R95–R99, were also reviewed and were included, according to certain epidemiological study evaluation methodologies for those cases (MONICA study protocol) [14]. The diagnosis of AMI was based on clinical data, instrumental examination data (electrocardiogram (ECG) changes), and an increase in cardiac-specific enzymes (alanine transaminase, aspartate transaminase, creatine kinase, troponin) in the blood, as well as on data from the pathologist’s anatomical and forensic medical examination [14].

For verification of AMI cases, four epidemiological diagnostic categories (EDCs) were set and coded as “definite AMI”, “possible AMI” (or, in case of death, “possible coronary death”), “not AMI” (or the cause of death was not coronary artery damage), and “insufficient data to define a category”. Cases with the following epidemiological diagnostic categories were included in the list of the study subjects: “definite AMI” and “possible AMI” (for survivors), and “definite AMI”, “possible coronary death”, and “insufficient data to define a category” (for patients who died) [15].

Every AMI event had to have its apparent onset within the study period and had to occur more than 28 days after any previously recorded AMI event in the same subject. Multiple AMI attacks occurring within 28 days of the onset of the symptoms of the first attack were considered as one event. An AMI event was defined as fatal if death occurred within the first 28 days from the onset. If the patient was alive after 28 days from the onset of the attack, the AMI was classified as non-fatal. All patients residing permanently in Kaunas city and suspected of having died from AMI or having had a non-fatal AMI were registered.

Mortality data were sourced from the Kaunas Civil Registry Office and the Lithuanian Cause of Death Register [16], with all medical death certificates reviewed and coded according to ICD-10 coding [17].

Fatal cases diagnosed with codes I20–I25 were selected and verified. The analysis focused on the individuals who died within 28 days of AMI onset. To ensure comprehensive identification of IHD-related mortality according to the WHO MONICA guidelines, additional ICD-10 codes were screened, including arterial hypertension (I10–I15), other heart diseases (I30–I52), cerebrovascular diseases (I60–I69), diseases of arteries, arterioles, and capillaries (I70–I77), and metabolic disorders, as diabetes, obesity and dyslipidemia (E10–E14, E65–E68, E78). Data consistency and comparability were maintained through standardized detection methods and diagnostic criteria throughout the entire study period.

### 2.2. Time of Hospitalization and Coronary Treatment

Information about the cases with AMI time of hospitalization and coronary care from 2000 to 2023 was obtained from the Kaunas IHD registry database. The study assessed the time of admission of individuals with AMI to the hospital: up to 2 h, from 2 to 6 h, from 6 to 24 h, and >24 h from the onset of pain.

The characteristics of treatment and reperfusion treatment, such as thrombolysis, percutaneous coronary angioplasty (PTCA), PTCA with stenting (PCI), and coronary artery bypass grafting (CABG) applied were also assessed.

During the 2000–2023 period, hospitalization time and coronary care volume data were evaluated in 6 study periods: 2000–2004, 2005–2008, 2009–2012, 2013–2016, 2017–2019, and 2020–2023.

The study included 8229 cases. The analysis showed that there were no missing values in the treatment variable groups. A total of 469 cases (an average 5.7%) were considered to be “missing” values for the hospitalization time variable over the period 2000–2023. “Missing” values for hospitalization time included categories of variables: new onset pain while staying in the hospital; iatrogenic pain (after a planned hospitalization, e.g., stenting or surgery); no pain (dyspnoea, weakness); treatment started from clinical death (resuscitation); and unclear cases. “Missing” values were excluded from hospitalization-time analyses because the timing variable was not classifiable.

Data were analyzed by sex and age group (25–54 and 55–64 years).

### 2.3. Statistical Analysis

Categorical variables were summarized by proportions expressed in percentages and compared by the chi-square test. Differences between two proportions were assessed using the z-test. Bonferroni correction was applied for multiple comparisons. A logistic regression analysis was performed to assess the odds ratios of death within 28 days across six time periods. The 2000–2004 period was used as the reference group. *p* value < 0.05 was considered statistically significant. The 28-day AMI case-fatality rates (in percentage) were calculated as a proportion of deaths to all AMI events multiplied by 100. The 28-day case-fatality rates from AMI were directly adjusted within 10-year age groups to the World Standard Population [18]. IBM SPSS Statistics for Windows, Version 28.0 was used for statistical analysis [19].

## 3. Results

In the period of 2000–2023, a total of 8229 cases of AMI were registered among Kaunas city residents aged 25–64, 74.5% of whom were men. Most cases of AMI were registered in the first study period (2000–2004), and the fewest cases were recorded in the period from 2017 to 2019.

Based on data obtained in the past two decades, changes in healthcare provision for patients with AMI, evaluated as hospitalization time from the onset of pain, were identified. The distribution of persons with AMI by time from the onset of pain to hospitalization in separate periods is presented in Table 1.

During the study period, the proportion of persons hospitalized up to 2 h from the onset of pain accounted for about one-fifth of all hospitalized patients from 2000 to 2016, while in the period from 2017 to 2023, it significantly decreased. A significant increase observed in 2009–2012, as compared to the previous period of 2005–2008, was followed by a decrease in the pre-pandemic period (2017–2019) and during the peri-COVID-19 pandemic, when compared to the previous time periods.

The proportion of patients hospitalized from 2 to 6 h after the pain onset significantly decreased in the periods of 2017–2019 and 2020–2023, compared to all the previous study periods. Consequently, the proportion of patients hospitalized from 6 to 24 h from the onset of pain significantly increased in the most recent observation years, compared to the periods evaluated in the 2000–2016-time interval. The proportion of AMI patients who were hospitalized later than 24 h after the onset of pain almost doubled in 2005–2008 and 2017–2019 compared to 2000–2004 (15.7%) and stayed elevated (28.3%) during the peri-COVID-19 pandemic period.

Based on data over the period from 2000 to 2023, it was found that on average, every fifth (21.3%) male who developed AMI was hospitalized within 2 h (Table 1). In 2017–2019 and 2020–2023, compared with the study periods of 2000–2004 and 2009–2016, significantly fewer men who developed AMI were hospitalized within the first 2 h of emergency presentation (*p* < 0.05).

The proportion of men hospitalized from 2 to 6 h due to AMI was the highest (30.6%) among all the men studied and varied significantly over the periods from the highest (39.9%) in 2000–2004 to the lowest of 21.6% (2017–2019) and 18.1% (2020–2023) (*p* < 0.05). Significant increase in proportions of men with AMI who were hospitalized in the time span from 6 to 24 h was observed in 2017–2019 and 2020–2023 (33.2% and 37.4%, respectively, *p* < 0.05), as well as the proportion of men who were hospitalized later than 24 h after the onset of pain (30.2% and 26.9%, respectively), when compared to previous study periods. In 2017–2019 and 2020–2023, when compared to previous study periods, a significant increase in proportions of men with AMI who were hospitalized in the time span from 6 to 24 h, and later than 24 h, was observed.

While comparing the results in sex groups (Table 1), it was identified that every sixth (16.0%) female with AMI was hospitalized up to 2 h over the last two study decades. The percentage of women who were hospitalized within 2 h from pain onset was significantly lower in 2017–2019 (8.5%) as compared to 2000–2004 (18.5%) (*p* < 0.05); this proportion stayed within the ranges of 13.6% to 18.5% with no significant differences over the remaining study periods. Over the whole study period, fewer women with AMI were hospitalized within the first 2 h of pain as compared to men (*p* < 0.05).

As well as in men, the proportion of women with AMI who were hospitalized from 2 to 6 h among all the women with AMI was the highest (29.3%). During the study period, this proportion of women decreased 3 times, from 34.1% (2000–2004) to 11.9% (2020–2023) (*p* < 0.05), while the proportion during the COVID-19 pandemic period did not significantly differ from that of pre-pandemic years (2017–2019). Significantly more women who developed AMI were hospitalized from 6 to 24 h after the emergency in 2017–2019, as compared to 2009–2012. Over the whole study period, the proportion of women with AMI who were hospitalized from 6 to 24 h was 28.3%, while on average, every fourth female was hospitalized later than 24 h from the beginning of pain.

The results obtained showed significant changes in the availability and application of therapy. The distribution of AMI patients according to reperfusion treatment methods in the study periods is presented in Table 2.

The end of the first decade marked a significant increase in the application of PCI and CABG when treating patients with AMI, as compared to the beginning of the study period (2000–2004), when reperfusion therapy was not applied to most of the AMI patients. The share of AMI patients not receiving reperfusion therapy decreased by about three times in 2020–2023 as compared to the initial study period. The application of PCI demonstrated a 30 times increase over the whole study years from 2000 to 2023, and the share of PCI increased from 2.2% to 61.9% (in 2013–2016) and stayed stable onwards, as well as during the COVID-19 pandemic period. The share of CABG reached the peak of 8.8% in 2005–2012, subsequently decreasing to the range of 4.6% (2017–2019) to 5.4% (2020–2023). The role of PTCA in the coronary care of the AMI patients in Kaunas appeared to be the greatest in the period of 2005–2008 (14.4%), subsequently diminishing to 0.1% in the time interval from 2017 to 2023 (*p* < 0.05). Thrombolysis remained a very rare (1.0–1.2%) treatment method due to the good availability of different reperfusion therapy capabilities, while in the period from 2017 to 2023, it was not applied at all.

The analysis of men revealed that a greater part of them have been put on reperfusion treatment since 2009–2012 (66.4%), and the proportion has been increasing during the pre-pandemic (72.4%) and peri-COVID-19 period (77.2%). PCI became the most frequently used reperfusion strategy since 2009. In men with AMI, PCI application has demonstrated stability from 2013 to 2023 (from 66.0% to 72.1%, respectively). The number of men who did not receive interventional reperfusion treatment has diminished by 3.6 times from 2000 to 2023. The application of PCI in men with AMI has increased almost 27 times during the study period. The proportion of PTCA applied in the treatment of AMI in men was the greatest in 2005–2008 and decreased to zero during the peri-COVID-19 pandemic. Thrombolysis was not the first-choice treatment during the study years; in 2000–2004, it was applied in 1.4% of AMI cases in men and was no longer used as of 2017.

Over the study period, fewer women received reperfusion treatment as compared to men (*p* < 0.05). Though the number of procedures applied was increasing from 2000 to 2023, the application of PCI has been significantly lower among women than in men during these years. Women with AMI tended to get less PTCA and CABG than men, while only thrombolysis was equally applied in both sexes during these decades. The pre-pandemic and peri-COVID-19 periods did not differ in proportions of reperfusion treatment methods applied in men and in women, while during 2017–2023, almost 4 women out of 10 did not receive reperfusion treatment at all.

The distribution of patients with AMI by time from the onset of pain to hospitalization in separate periods in age groups is presented in Table 3.

Data indicated that from 2000 to 2023, on average, almost every fifth 25–54-year-old AMI patient (21.6%) was hospitalized within the first 2 h of the beginning of pain. This proportion of patients stayed stable in the pre-pandemic and peri-COVID-19 pandemic periods.

Most of the 25–54-year-old people (29.0%) were hospitalized within 2 to 6 h of the emergency. The proportion of AMI patients arriving at the hospital within this time interval from the start of pain has significantly diminished in 2017–2019 and 2020–2023, as compared to 2000–2004 and 2013–2016. In 2017–2023, a significant increase in the number of patients hospitalized for 6 to 24 h was observed, in comparison to the previous periods from 2000 to 2016. The proportion of AMI patients who were hospitalized later than 24 h from the onset of emergency was rather stable throughout 2005–2023 and demonstrated no significant changes during the peri-COVID-19 pandemic in this age group.

The analysis of the older age group revealed that every fifth (18.9%) person aged 55–64 who developed AMI was hospitalized within 2 h from the onset of pain; this proportion has decreased significantly in 2017–2019 (11.8%) as compared to the previous time periods. Significant differences in times to hospitalization in 2017–2023 were observed when compared to most of the previous study periods, but there were no significant differences in all patient hospitalization times (in hours) intervals between pre-pandemic (2017–2019) and peri-COVID-19 pandemic (2020–2023) periods.

There were no significant differences in times from the onset of pain to hospitalization between both age groups, with the exception of 2009–2012, when more people with AMI from the younger age group were hospitalized within the first 2 h (*p* < 0.05) than from the 55–64-year-old age group.

The results from the distribution of patients with AMI by interventional treatment methods in separate periods in age groups (Table 4) showed that the percentage of patients who did not receive reperfusion treatment has decreased three times in 2020–2023 as compared to the initial period of 2000–2004, possibly due to better patient selection and availability of reperfusion therapy.

Among all the reperfusion therapies applied in both age groups, most often PCI was performed. In the latest study period, PCI treatment was performed 23 times more frequently in younger patients and 42 times more frequently in older patients than at the beginning of the study.

There were significant differences between the age groups until 2017, when PCI (2000–2016) as well as CABG (2009–2016) were more prevalent reperfusion therapies among the 25–54-year-old AMI patients (*p* < 0.05). From 2017 to 2023, most of the AMI patients in both age groups received PCI, showing no differences between the younger and the older age groups in the reperfusion procedures applied, as well as no differences between treatment in these groups during the pre-pandemic and peri-COVID-19 periods.

The in-hospital case-fatality rate from AMI with 95% confidence intervals among Kaunas men and women aged 25–64 years by study period and sex is presented in Table 5. It was found that over the 6 study periods, these rates varied from 2.4 to 10.6% for men, and from 4.2 to 8.1% for women. The in-hospital case-fatality rate for men in the 2017–2019 period was significantly lower compared to the other study periods, while it did not differ significantly for women.

The odds ratio to die within 28 days after AMI among Kaunas men aged 25–64 years by study period (logistic regression analysis) is presented in Table 6.

As compared to the 2000–2004 period, the chance of dying within 28 days of AMI in the 2009–2012 study period was significantly higher on average by 1.4 times (95% CI 1.04–1.9), while in the 2017–2019 and 2020–2023 periods, it was significantly lower on average, by 69% and 34%, respectively (95% CI 0.17–0.55 and 95% CI 0.44–0.98).

The odds ratio of dying within 28 days after AMI among Kaunas women aged 25–64 years by study period (logistic regression analysis) is presented in Table 7.

In the periods of 2005–2008 and 2017–2019, the odds of dying within 28 days after the onset of AMI for women were significantly lower on average, by 61% and 73%, respectively (95% CI 0.19–0.77 and 95% CI 0.08–0.89), compared to the period of 2000–2004.

## 4. Discussion

The study data from the Kaunas IHD Registry represent an overview of the management of AMI and its changes over the past two decades; at the same time, it indirectly provides a reflection of changes in the healthcare system in the whole country during the period from 2000 to 2023.

At the beginning of the study period in 2000, Lithuania’s healthcare system was in transition, attempting to increase the efficiency of health services. Decentralization and privatization have been implemented as part of its policy to achieve greater efficiency. The restructuring of the healthcare system in the country was balancing between decentralization and centralization, and between public and private healthcare sectors [20]. In 1998, the major causes of mortality in Lithuania were diseases of the circulatory system (standardized rate of 5.28 per 1000 population) [21]. In 2000, the European Observatory on Health Care Systems indicated that about 95% of the Lithuanian population lived within 20 km of the nearest general hospital and within 120 km of the nearest regional or university hospital, and access to hospital care was quite good [21]. However, there were concerns regarding the quality of care provided and the financial resources required to maintain the hospital network of that time; thus, reorganization of the inpatient network has been placed on the agenda for reforms and renovation of facilities became one of the targets of national investment policy [21], which led to the reforms and development of the healthcare system in the first two decades of the 21st century.

Our study took place in the second largest city in Lithuania, during a period when the reforms in the country’s healthcare system underwent major efforts to decrease the number of inpatient active treatment cases and create a more efficient healthcare system providing quality healthcare services for the population. It is important to admit that the economic development of the country over the last two decades has also changed, and since 2013, Lithuania has been classified in the high-income category group [22].

Lithuania became a full member of the European Union (EU) on 1 May 2004. It stands out from other EU countries with obvious health inequalities between rural and urban people, with different education, family, economic status, and other population groups. For example, the standardized mortality rate of rural residents in 2004–2013 was on average 22.4% higher than that of urban residents, and in 2013, the average life expectancy of urban residents was 2.7 years longer. These inequalities are determined by many reasons—different education levels, economic status, and accessibility of healthcare services between urban and rural residents [23].

Requirements and quality indicators of provision of personal healthcare services in suspected or diagnosed AMI with and without STEMI established in Lithuania [24] have been monitored and subsequently revised [24,25,26]. In 2014, integrated healthcare services (clusters), among which the AMI cluster has been introduced, where Kaunas city plays a key role in the region as a PCI center [27] in the territories where regional functional healthcare is provided [28].

It should be noted that the Cardiology cluster hospitals operate 24/7 for all the patients with AMI who can be treated with invasive therapy, while these AMI patients who cannot be treated with invasive therapy because of clinical contraindications are usually referred and hospitalized at non-Cardiology cluster hospitals, where they receive non-invasive treatment or thrombolysis.

Between 2013 and 2023, there were declines in the standardized death rates of several leading causes of death in the EU: circulatory diseases decreased by 23.2%, deaths from IHD dropped by 25.6%, and heart attacks declined by 35.3% [29].

Since 2000, the inpatient hospital network in Kaunas city has been undergoing reorganizational, structural changes; this may partially explain the observed decrease in hospitalization rate (13.7%) of patients with AMI within the first 2 h of pain onset in the pre-pandemic period (2017–2019) compared to the previous time periods of 2009–2012 (22.8%) and 2013–2016 (21.7%) (*p* < 0.05).

In 2022, the Development Program for Improving the Quality and Efficiency of Healthcare in Lithuania 2022–2030 admitted the need to address high mortality from preventable diseases. Among the causes of the problem to be addressed in CVD healthcare provision, the cardiology cluster, which did not ensure the possibility of receiving quality services on time, is inefficient in emergency medical service management [30,31,32,33,34]. In 2022, the Health Protection and Promotion Development Program of the Ministry of Health was also approved. It is intended to develop public healthcare, which includes a set of organizational, legal, economic, technical, social, and medical measures that help implement disease and injury prevention, preserve and strengthen public health [35]. Progress measure result indicators, such as the share of emergency medical services provided within 15 and 25 min, in urban and rural areas, respectively, have been approved to monitor progress from the initial year to 2030 [35]. On 1 July 2023, the new National Emergency Medical Service began operating, uniting 48 independent emergency medical service stations operating in Lithuania [36]. Study findings indicate that the availability of hospital services within the first hours of the emergency has not significantly deteriorated during the COVID-19 pandemic period for all patients. As evaluated in 2023, the accessibility to healthcare services in Kaunas County (% of population living within 15 min of a hospital, by NUTS 3 regions) was 86.8% [37].

Over the past two decades, prolonged hospitalization times among patients with AMI have remained a persistent problem despite advances in cardiology. Studies indicate that these delays are multifactorial and largely occur before contact with the healthcare system. Patient decision time is considered the major contributor, accounting for up to 75% of the total delay [38]. Older age and multimorbidity are also associated with delayed hospitalization, particularly among patients with arterial hypertension (AH), diabetes, and previous myocardial infarction (MI) [39]. In addition, atypical AMI presentations, such as dyspnea, nausea, or fatigue instead of chest pain, are more common in women and elderly patients and may delay symptom recognition [38,40]. Psychological and behavioral factors, including waiting for symptoms to resolve and contacting family physicians instead of emergency services, further prolong delays [38]. Population-level hospitalization delays have shown little improvement over time, while the increasing prevalence of NSTEMI and atypical AMI presentations has complicated timely diagnosis and treatment [39].

In our study, a higher proportion of AMI patients were hospitalized more than two hours after symptom onset during the last two study periods. This may be explained by a greater prevalence of comorbidities such as AH, diabetes, and obesity, particularly among older individuals. The unpublished Registry data over the last two decades has shown an increase in the prevalence of AH by 30–50%, obesity—from 3 to 4 times, and diabetes—2 times, among persons with AMI in the age group of 55–64 years, in particular, both in men and women. In our study, among individuals with AMI, the proportion of cases with a different or unclear onset of the disease over the last two periods increased twice compared to the remaining study periods, which could have influenced the delayed time to hospitalization. Finally, reduced access to healthcare services during the COVID-19 pandemic may also have contributed to delayed hospitalization [41].

Since early recognition and treatment of a heart attack is critical to the outcome, the awareness of patients about the symptoms of the onset of coronary acute emergency in Lithuania has been improved by population education and involvement in preventive programs [42,43,44]. The number of program participants has been consistently increasing over the period of 2015–2025, the Summary Report of the Institute of Hygiene (2023) [45] on the evaluation of the effectiveness of the ongoing Health Promotion Program for Prevention of Cardiovascular Diseases and Diabetes in Lithuania (approved by the Ministry of Health of Lithuanian Republic in 2014, subsequently revised) indicated that from 2015 through 2023 the number of program graduates has been growing by 28% [44,46].

Our findings revealed no differences in times from the onset of pain to hospitalization in both age groups in 2000–2008 and 2013–2023, while between 2009 and 2012, more 25–54-year-old AMI patients were admitted within the first 2 h than in the 55–64-year-old age group (*p* < 0.05). The number of urban Kaunas residents aged 25–64 years receiving coronary reperfusion treatment has significantly increased over the past two decades and has demonstrated no differences in the age groups since 2017. It was observed that in 2017–2023, significantly fewer men who developed AMI were hospitalized within the first 2 h from the onset of pain. Throughout the study period, fewer women with AMI were hospitalized within the first two hours of pain as compared to men (*p* < 0.05).

Studies from other countries indicated that women often wait longer than men after experiencing symptoms to call for help; older age, female sex, and symptom onset at night were all associated with a longer delay to call for emergency medical services and that these were synergistic, with a much greater delay at night, for older, female patients than for younger women, or for men of any age, and a greater effect of age on time for call for female patients than male patients [47,48]. A study from Italy found that women with AMI face higher mortality largely due to older age and undertreatment during hospitalization and after discharge [49].

Improvements in the prevalence of CVD risk factors, treatment, and control rates have shown positive tendencies in Eastern European countries over the first decades of the 21st century [50,51,52].

In a developing country, Kosovo, STEMI was more frequent than non-ST-elevated myocardial infarction (NSTEMI), affecting younger male patients; the leading risk factors included arterial hypertension, smoking, diabetes mellitus, and a family history of CVD [53]. The decline in acute MI-related mortality over recent years can be explained by the increasing use of myocardial reperfusion procedures [53]. While data from Belgium indicates that over 30 years, AMI incidence, particularly STEMI, decreased threefold across all age groups and genders, while NSTEMI incidence initially declined but increased after 2000 due to improved diagnostic sensitivity with troponins, and survival rates improved with greater use of angiography and revascularization [54]. A cohort study from Iceland states that the change in population-level risk factor exposure is likely to have influenced atherosclerotic plaque burden and thrombotic mechanisms, and increasing uptake of cardioprotective pharmacological and interventional therapy may have resulted in a primary preventive effect on plaque rupture and thrombosis and thus on the rates of STEMI and NSTEMI disproportionally [55].

With an increasing share of the aging population, Lithuanian people experience the challenges of extended lifetime, such as functional decline and chronic diseases [48,56], leading to increased risks of CVD and unrecognized AMI as well. The prevalence of primary arterial hypertension in middle-aged Lithuanians was high, reaching almost 50% in both sexes. Patients tended to have many cardiovascular risk factors simultaneously, with dyslipidemia being the most common (prevalence > 90%) [57].

AMI that remains undetected in the acute phase is associated with an unfavorable prognosis; individuals with unrecognized AMI are characterized by a substantial burden of metabolic risk factors, which might lead to insufficient recognition and management of CVD risk [58]. Multivessel disease also represents a clinical challenge in patient management and decision making, with several areas of uncertainty, such as the optimal timing for complete revascularization or the best guiding strategy for intermediate stenoses [59,60].

PCI is the internationally preferred therapy for ST-segment elevation myocardial infarction (STEMI) [13]. The Irish Heart Attack Audit National Report in 2024 revealed that only 77% of patients with a STEMI received primary PCI, compared with 86% in 2017 [61]. Long-term surgical outcomes of postinfarction mechanical complications remain understudied [62]. In ISCHEMIA, both PCI and CABG were associated with better 3-year health status than conservative management; better angina relief with CABG than PCI was seen at 1, but not 3, years [63].

Recent analysis revealed that during 2000–2023, the age-standardized AMI morbidity among urban 25–64-year-old Kaunas residents significantly decreased, while the age-standardized mortality from IHD significantly decreased among men only [64].

This study demonstrates two contrasting temporal trends in the management of AMI over the past two decades in Kaunas city: a marked improvement in the availability and use of reperfusion therapy, particularly PCI, alongside a paradoxical shift toward longer pre-hospital delays. In recent years, a significantly smaller proportion of patients—especially women—presented within the first 2 h, while late presentations (6–24 h and >24 h) increased. Although sex and age differences in treatment have narrowed over time, women consistently remain less likely to receive timely hospitalization and reperfusion therapy. These findings suggest that, despite substantial advancements in coronary care, patient-related delays and potential differences in symptom recognition, health-seeking behavior, and social factors continue to play a critical role in determining access to effective treatment.

Measurement of a biomarker of cardiomyocyte injury, preferably high-sensitivity cardiac troponin (hs-cTn), is recommended by the guidelines of the European Society of Cardiology in all patients with suspected acute coronary syndrome [11]. In the last decade, troponin hs-cTn and N-terminal pro-brain natriuretic peptide (NT-proBNP) gained a significant role in cardiology. Both troponin TnT and NT-proBNP measurements exhibit prognostic abilities in critically ill patients as nonspecific markers of volume overload and myocardial ischemia. The study by Lenz m. et al. on NT-proBNP and troponin hs-cTn exhibited additive prognostic value for the outcome of critically ill patients [65].

Over the past few decades, advances in pharmacological, catheter-based, and surgical reperfusion have improved outcomes for patients with AMI; however, patients with large infarcts or those who do not receive timely revascularization remain at risk for mechanical complications of AMI. The most encountered mechanical complications are acute mitral regurgitation secondary to papillary muscle rupture, ventricular septal defect, pseudoaneurysm, and free wall rupture; each complication is associated with a significant risk of morbidity and mortality [66]. The care for patients with mechanical complications is complex and requires a multidisciplinary collaboration for recognition, diagnosis, hemodynamic stabilization, and decision support, though a significant variability in care exists, which mainly depends on local expertise and available resources. It should also be noted that mechanical complications are often part of subacute myocardial infarction and more often occur in the group of patients who delay seeking emergency medical services.

With the introduction of the ST-segment elevation myocardial infarction cluster regulations in 2014 and the entry into force of the non-ST-segment elevation MI cluster regulations, Kaunas AMI cluster hospital (Cardiology Center) accepts patients transferred from other Kaunas city and county hospitals with AMI for urgent coronary angiography around the clock. A multidisciplinary “Heart Team” (cardiologists, cardiac surgeons, radiologists, vascular surgeons, anesthesiologists, neurologists, endocrinologists, nephrologists, or other specialists, selecting the best possible treatment tactics) approach has been implemented.

Our analysis revealed that from 2000 to 2023, the prevalence of arterial hypertension among persons with AMI has increased by 30–50%, obesity increased from 3 to 4 times, and diabetes increased 2 times in the age group of 55–64 years, particularly in both men and women. These findings might only partially explain the possible reasons for the delayed time from pain onset to hospitalization, while other possible individual factors (patient decision time, atypical or less intense symptoms, etc.), organizational factors (care delays, time to admission, etc.) and the COVID-19 pandemic could add more explanatory information.

Real-world data analysis from another AMI cluster hospital in Lithuania [67] revealed that in-hospital mortality due to AMI as the main cause of death in 2012–2017 compared to 2006–2011 decreased by 9.0% (from 25.6% to 16.6%), higher case-fatality rates were due to severe, acute and chronic concomitant diseases, hyper diagnostics of AMI, while the observed positive changes in later years were due to improved diagnostics, treatment of AMI and serious concomitant diseases.

A recent study in Norway indicated that thirty-day case fatality for all types of MI in total was 21.3% in 2013 and 17.5% in 2021; case fatality for all MI fell by an average of 2.8% per year (95% CI 2.3–3.3), case fatality for NSTEMI fell by 4.4% per year (95% CI 3.3–5.5) per year, while case fatality for STEMI was unchanged [68].

Our study findings revealed the in-hospital case-fatality rate for men in the 2017–2019 and 2020–2023 periods. Compared to the period 2000–2004, the odds of dying within 28 days from AMI were significantly lower for men from 2017, but for women, these odds were insignificant from 2020. As cardiovascular medical therapy has undergone major changes in Kaunas during the past two decades, part of the temporal evolution in the outcomes and care patterns likely reflects advances in background medical therapy. Innovative medical devices and technologies are revolutionizing CVD management, facilitating early diagnosis, continuous monitoring, and effective treatment, improving patient outcomes and reducing healthcare costs.

Optimization of acute coronary syndrome management and optimal reperfusion service protocol for AMI by standardizing care in sex and age groups could be beneficial in reducing the burden of CVD in Kaunas city residents aged 25–64 years, alongside the ongoing measures provided across the country. To provide better coronary care services for AMI patients, further detailed studies on the processes involved are ongoing.

### Strengths and Limitations of the Study

Our study has several limitations. First, it is a retrospective analysis, which depends on the accuracy of the data provided in medical statistical records, the accuracy of coding of the time of the onset of pain in AMI patients, as well as of the AMI events. Second, we cannot exclude the possibility that our results over the study period could be influenced by incorrect coding, misclassification, or misdiagnosis, especially in women, who are older in age, and with SARS-CoV-2 infection in the last study period during the COVID-19 pandemic. Third, over the last few decades, diagnostic and treatment methods for persons with AMI have changed substantially, which depended on the time of admission of patients to the hospital, and these changes were not assessed in our study. Troponin and NT-proBNP levels were not included in our study analysis due to changes in laboratory assays over time, which limited their comparability across the study period and represent a methodological limitation. Fourth, our study did not assess the comorbidities of persons with AMI, which could also have determined both the time of hospitalization and the choice of a possible further treatment method. Fifth, the definitions of clinical AMI diagnosis over the 2000–2023 period evolved substantially, with the implementation of successive Universal Definitions of MI, increasing reliance on cardiac troponin measurements, and improved differentiation between STEMI and NSTEMI. A limitation of the study is that we did not assess AMI types, although treatment options and hospitalizations may have been influenced by new diagnostic methods and STEMI or NSTEMI type. Apart from the ongoing healthcare system reform, improvements and subsequent local organizational changes, some individual and procedural aspects were not analyzed, which is a limitation for this study. Lastly, in our study, the length of hospital stays, which could potentially be related to the possibility of applying one or another treatment procedure, was not analyzed, and this would be one of the limitations of the study.

## 5. Conclusions

In Lithuania, among urban patients with AMI, coronary care has changed significantly over the past two decades. One-fifth of all hospitalized patients were admitted to the hospital within the first few hours from the onset of pain, while in the peri-COVID-19 period, this decreased. The number of PCIs has increased significantly due to new emergency medical care algorithms, an increasing number of centers, and, consequently, better connectivity with main PCI centers. The number of thrombolytic procedures in Kaunas has been relatively low, and the percentage of CABG has not changed significantly, as tailored therapeutic decisions that balance ischemic benefit against procedural risk were taken into account. Male patients with AMI underwent PCI more often than women. During several study periods, CABG was performed more often in the older AMI patients than in the younger ones. Compared to the period of 2000–2004, the odds of dying within 28 days from AMI were significantly lower for men and women from 2017, but for women, these odds were insignificant from 2020.

## Figures and Tables

**Table 1:** Distribution (%) of patients with acute myocardial infarction by time from the onset of pain to hospitalization in the study periods.

Time from the Onset of Pain to Hospitalization	Period (Years)					
	2000–2004	2005–2008	2009–2012	2013–2016	2017–2019	2020–2023
	Total					
	*n* = 1979	*n* = 1542	*n* = 1434	*n* = 1118	*n* = 767	*n* = 920
Up to 2 h *	22.1	18.2	22.8 ^b^	21.7	13.7 ^a,c,d^	16.8 ^a,c^
From 2 to 6 h	38.4	29.2 ^a^	30.8 ^a^	34.0	21.3 ^a,b,c,d^	17.0 ^a,b,c,d^
From 6 to 24 h	23.9	24.5	22.6	23.2	33.9 ^a,b,c,d^	37.9 ^a,b,c,d^
Later than 24 h	15.7	28.1 ^a^	23.8 ^a^	21.1 ^a,b^	31.2 ^a,c,d^	28.3 ^a,d^
	Men					
	*n* = 1443	*n* = 1105	*n* = 1033	*n* = 859	*n* = 603	*n* = 744
Up to 2 h	23.4	19.7	24.6	23.2	15.1 ^a,c,d^	17.6 ^a,c,d^
From 2 to 6 h	39.9	29.1 ^a^	30.0 ^a^	34.7	21.6 ^a,b,c,d^	18.1 ^a,b,c,d^
From 6 to 24 h	21.6	24.2	22.7	22.4	33.2 ^a,b,c,d^	37.4 ^a,b,c,d^
Later than 24 h	15.1	27.0 ^a^	22.7 ^a^	19.8 ^b^	30.2 ^a,c,d^	26.9 ^a,d^
	Women					
	*n* = 536	*n* = 437	*n* = 401	*n* = 259	*n* = 164	*n* = 176
Up to 2 h	18.5	14.2	18.2	17.0	8.5 ^a^	13.6
From 2 to 6 h	34.1	29.3	32.9	31.7	20.1 ^a,c,d^	11.9 ^a,b,c,d^
From 6 to 24 h	30.0	25.4	22.2	25.9	36.6 ^c^	40.3 ^b,c,d^
Later than 24 h	17.4	31.1 ^a^	26.7 ^a^	25.5	34.8 ^a^	34.1 ^a^

* Data are presented as percentages. Superscript letters indicate statistically significant differences between study periods after Bonferroni correction for multiple comparisons (15 pairwise comparisons): ^a^ *p* < 0.05 vs. 2000–2004; ^b^ *p* < 0.05 vs. 2005–2008; ^c^ *p* < 0.05 vs. 2009–2012; ^d^ *p* < 0.05 vs. 2013–2016.

**Table 2:** Distribution (%) of patients with acute myocardial infarction in sex groups according to reperfusion treatment methods used in the study periods.

Reperfusion Treatment	Period (Years)					
	2000–2004	2005–2008	2009–2012	2013–2016	2017–2019	2020–2023
	Total					
	*n* = 2059	*n* = 1591	*n* = 1487	*n* = 1156	*n* = 854	*n* = 1082
Not applied *	84.0	53.4 ^a^	42.5 ^a,b^	30.9 ^a,b,c^	30.8 ^a,b,c^	26.8 ^a,b,c^
Thrombolysis	1.2	0.2 ^a^	0.2 ^a^	0.7	0.0 ^a^	0.0 ^a^
PTCA	7.8	14.4 ^a^	1.9 ^a,b^	0.6 ^a,b^	0.1 ^a,b,c^	0.1 ^a,b,c^
PCI	2.2	23.2 ^a^	46.7 ^a,b^	61.9 ^a,b,c^	64.5 ^a,b,c^	67.7 ^a,b,c^
CABG	4.8	8.8 ^a^	8.7 ^a^	6.0	4.6 ^b,c^	5.4 ^b,c^
	Men					
	*n* = 1498	*n* = 1137	*n* = 1069	*n* = 886	*n* = 678	*n* = 868
Not applied	81.7	47.5 ^a^	33.6 ^a,b^	25.7 ^a,b,c^	27.6 ^a,b^	22.8 ^a,b,c^
Thrombolysis	1.4	0.3	0.3	0.7	0.0	0.0
PTCA	8.6	16.5 ^a^	2.4 ^a,b^	0.7 ^a,b,c^	0.1 ^a,b,c^	0.0 ^a,b,c^
PCI	2.7	26.2 ^a^	53.8 ^a,b^	66.0 ^a,b,c^	67.4 ^a,b,c^	72.1 ^a,b,c^
CABG	5.6	9.5 ^a^	9.9 ^a^	6.9	4.9 ^b,c^	5.1 ^b,c^
	Women					
	*n* = 561	*n* = 454	*n* = 418	*n* = 270	*n* = 176	*n* = 214
Not applied	90.2	68.3 ^a^	65.3 ^a^	47.8 ^a,b,c^	43.2 ^a,b,c^	43.0 ^a,b,c^
Thrombolysis	0.5	0.0	0.0	0.7	0.0	0.0
PTCA	5.7	9.0	0.5 ^a,b^	0.4 ^a,b^	0.0 ^a,b^	0.5 ^a,b^
PCI	0.9	15.6 ^a^	28.7 ^a,b^	48.1 ^a,b,c^	53.4 ^a,b,c^	50.0 ^a,b,c^
CABG	2.7	7.0 ^a^	5.5	3.0	3.4	6.5

* Data are presented as percentages. Superscript letters indicate statistically significant differences between study periods after Bonferroni correction for multiple comparisons (15 pairwise comparisons): ^a^ *p* < 0.05 vs. 2000–2004; ^b^ *p* < 0.05 vs. 2005–2008; ^c^ *p* < 0.05 vs. 2009–2012; PTCA—percutaneous coronary angioplasty; PCI—PTCA with stenting; CABG—coronary artery bypass grafting.

**Table 3:** Distribution (%) of patients with acute myocardial infarction in age groups by time from the onset of pain to hospitalization in separate periods.

Time from the Onset of Pain to Hospitalization	Period (Years)					
	2000–2004	2005–2008	2009–2012	2013–2016	2017–2019	2020–2023
	Age 25–54 years					
	*n* = 781	*n* = 582	*n* = 540	*n* = 416	*n* = 259	*n* = 287
Up to 2 h *	22.7	19.6	26.1	22.4	17.4	17.4
From 2 to 6 h	38.8	27.3 ^a^	27.6 ^a^	30.8	18.5 ^a,d^	15.7 ^a,b,c,d^
From 6 to 24 h	22.7	25.3	22.4	22.8	36.3 ^a,b,c,d^	35.9 ^a,b,c,d^
Later than 24 h	15.9	27.8 ^a^	23.9 ^a^	24.0 ^a^	27.8 ^a^	31.0 ^a^
	Age 55–64 years					
	*n* = 1198	*n* = 960	*n* = 894	*n* = 702	*n* = 508	*n* = 633
Up to 2 h	21.7	17.3	20.8	21.4	11.8 ^a,c,d^	16.6
From 2 to 6 h	38.1	30.3 ^a^	32.8	35.9	22.6 ^a,b,c^.^d^	17.5 ^a,b,c,d^
From 6 to 24 h	24.6	24.1	22.7	23.4	32.7 ^a,b,c,d^	38.9 ^a,b,c,d^
Later than 24 h	15.6	28.3 ^a^	23.7 ^a^	19.4 ^b^	32.9 ^a,c,d^	27.0 ^a,d^

* Data are presented as percentages. Superscript letters indicate statistically significant differences between study periods after Bonferroni correction for multiple comparisons (15 pairwise comparisons): ^a^ *p* < 0.05 vs. 2000–2004; ^b^ *p* < 0.05 vs. 2005–2008; ^c^ *p* < 0.05 vs. 2009–2012; ^d^ *p* < 0.05 vs. 2013–2016.

**Table 4:** Distribution (%) of patients with acute myocardial infarction in age groups according to reperfusion treatment methods used in the study periods.

Reperfusion Treatment	Period (Years)					
	2000–2004	2005–2008	2009–2012	2013–2016	2017–2019	2020–2023
	Age 25–54 years					
	*n* = 817	*n* = 600	*n* = 556	*n* = 425	*n* = 280	*n* = 333
Not applied *	83.2	49.5 ^a^	38.7 ^a,b^	26.1 ^a,b,c^	30.0 ^a,b^	26.1 ^a,b,c^
Thrombolysis	1.5	0.2	0.0	0.2	0.0	0.0
PTCA	8.4	14.8 ^a^	1.8 ^a,b^	0.5 ^a,b^	0.4 ^a,b^	0.0 ^a,b^
PCI	3.1	28.3 ^a^	54.5 ^a,b^	69.6 ^a,b,c^	66.8 ^a,b,c^	69.7 ^a,b,c^
CABG	3.8	7.2	5.0	3.5	2.9	4.2
	Age 55–64 years					
	*n* = 1242	*n* = 991	*n* = 931	*n* = 731	*n* = 574	*n* = 749
Not applied	84.5	55.8 ^a^	44.8 ^a,b^	33.7 ^a,b,c^	31.2 ^a,b,c^	27.1 ^a,b,c^
Thrombolysis	1.0	0.2	0.3	1.0	0.0	0.0
PTCA	7.4	14.1 ^a^	1.9 ^a,b^	0.7 ^a,b^	0.0 ^a,b,c^	0.1 ^a,b,c^
PCI	1.6	20.1 ^a^	42.1 ^a,b^	57.3 ^a,b,c^	63.4 ^a,b,c^	66.9 ^a,b,c,d^
CABG	5.5	9.8 ^a^	10.8 ^a^	7.4	5.4 ^b,c^	5.9 ^b,c^

* Data are presented as percentages. Superscript letters indicate statistically significant differences between study periods after Bonferroni correction for multiple comparisons (15 pairwise comparisons): ^a^ *p* < 0.05 vs. 2000–2004; ^b^ *p* < 0.05 vs. 2005–2008; ^c^ *p* < 0.05 vs. 2009–2012; ^d^ *p* < 0.05 vs. 2013–2016; PTCA—percutaneous coronary angioplasty; PCI—PTCA with stenting; CABG—coronary artery bypass grafting.

**Table 5:** In-hospital case-fatality rates (%) with 95% confidence intervals after acute myocardial infarction among Kaunas men and women aged 25–64 years by study periods and sex.

Sex	Study Periods					
	2000–2004	2005–2008	2009–2012	2013–2016	2017–2019	2020–2023
Men	7.44 (4.97–9.41)	8.36 (5.76–10.42)	10.63 (7.72–12.92)	10.01 (6.97–12.36)	2.39 * (0.65–3.75)	4.39 (1.63–6.44)
Women	6.79 (2.42–9.77)	4.92 (1.03–7.60)	7.65 (3.09–10.74)	8.13 (2.27–11.72)	4.20 (−0.31–7.01)	6.02 (−0.50–9.45)

* *p* < 0.05 compared with other periods.

**Table 6:** Odds ratio to die within 28 days after acute myocardial infarction among Kaunas men aged 25–64 years by study periods (logistic regression analysis).

Time Period	B	Exp (B)	95% CI		*p* Value
			Lower	Upper	
2000–2004 *					
2005–2008	−0.192	0.826	0.587	1.161	0.271
2009–2012	0.339	1.403	1.035	1.904	0.029
2013–2016	0.146	1.157	0.826	1.619	0.396
2017–2019	−1.185	0.306	0.170	0.551	0.000
2020–2023	−0.420	0.657	0.441	0.980	0.040

* reference period.

**Table 7:** Odds ratio to die within 28 days after acute myocardial infarction among Kaunas women aged 25–64 years by study periods (logistic regression analysis).

Time Period	B	Exp (B)	95% CI		*p* Value
			Lower	Upper	
2000–2004 *					
2005–2008	−0.955	0.385	0.193	0.768	0.007
2009–2012	−0.150	0.861	0.496	1.495	0.595
2013–2016	−0.092	0.912	0.488	1.705	0.772
2017–2019	−1.314	0.269	0.082	0.886	0.031
2020–2023	−0.083	0.921	0.468	1.814	0.811

* reference period.

## Data Availability

The original contributions presented in this study are included in the article; further inquiries can be directed to the corresponding authors.

## Abbreviations

The following abbreviations are used in this manuscript:

AMI	Acute myocardial infarction
IHD	Ischemic heart disease
CABG	Coronary aorta bypass grafting
NSTEMI	Non-ST-elevated myocardial infarction
STEMI	ST-elevated myocardial infarction
PTCA	Percutaneous coronary angioplasty
PCI	Percutaneous coronary angioplasty with stenting
